# A cursed duet—a case of tuberculous effusive-constrictive pericarditis

**DOI:** 10.1007/s11845-025-04013-3

**Published:** 2025-07-29

**Authors:** Jack Hartnett, Niall Connolly, Ross Murphy, Andrew O. Maree

**Affiliations:** 1 Department of Cardiology, St James’s Hospital, James’s Street, Dublin 8, Ireland; 2 School of Medicine, Trinity College Dublin, Dublin, Ireland

**Keywords:** Cardiac tamponade, Constrictive pericarditis, Effusive constrictive pericarditis

## Abstract

**Introduction:**

Effusive constrictive pericarditis (ECP) is a syndrome of combined pericardial effusive and constrictive physiology. Historically, it was a rare diagnosis which required invasive cardiac catheterisation confirmation post drainage of the pericardial effusion. However, the emergence of reliable echocardiographic features of constrictive disease has improved diagnostic yield.

**Case:**

We present a case of a 34-year-old patient with a pericardial effusion secondary to tuberculous pericarditis which demonstrated constrictive features on echocardiogram pre-pericardiocentesis and on invasive cardiac catheterisation measurements post pericardiocentesis – consistent with ECP. Ultimately this patient required a pericardiectomy for refractory symptoms of right heart failure despite pericardiocentesis.

**Discussion:**

We utilise this case to highlight the rare clinical entity of ECP but also to demonstrate the complex haemodynamics which govern pericardial effusions, constrictive pericarditis and ECP.

## Introduction

Effusive-constrictive pericarditis (ECP) is a syndrome of combined pericardial effusive and pericardial constrictive physiology. It is diagnosed haemodynamically by persistently raised right atrial (RA) pressure (> 10 mm Hg or drop of < 50%) despite relief of the raised intra-pericardial pressure via pericardiocentesis.

ECP is uncommon, and thus, data is lacking. In the largest ECP study to date, involving 1184 pericarditis patients between 1986 and 2001 (of which 190 underwent combined pericardiocentesis and cardiac catheterisation), 15 were eventually diagnosed with ECP [1]. The underlying aetiologies of ECP were diverse—7 idiopathic, 4 neoplastic, 1 post-surgical, 1 tuberculous, and 2 radiation induced. Looking more specifically at constrictive pericarditis patients who underwent pericardiectomy, ECP occurred in 24% overall with broadly equal prevalence rates for all aetiologies [2]. In an undifferentiated cohort undergoing pericardiocentesis, ECP was diagnosed in 16% [3]. Beyond these relatively small retrospective studies, data on ECP is limited to case reports.

Here, we present a rare case of ECP secondary to tuberculous pericarditis. It acts as a useful teaching case to demonstrate the complex haemodynamics underlying ECP, constrictive pericarditis, and pericardial effusions more broadly.

## Case

A 34-year-old male presented to the Emergency Department (ED) with a 3-day history of feeling generally unwell, febrile and fatigued. He denied any shortness of breath, chest pain or night sweats.

His past medical history was significant for latent tuberculosis (TB) treated with 9 months of Isoniazid therapy 2 years earlier. He denied blood-borne virus infection with the most recent negative human immunodeficiency virus (HIV) test approximately 6 years prior.

On examination, he was febrile (38 °C) and tachycardic (118 bpm) but not hypotensive (117/74 mm Hg). His jugular venous pressure (JVP) was measured at 10 cm. Pulse was regular. Heart sounds were dual with nil added but notably distant. Auscultation of lung fields yielded decreased air entry with bi-basal crackles.

Laboratory investigations revealed a normocytic anaemia (Hb 11.2, MCV 82), hyponatraemia (Na 125) and a transaminitis (ALT 119 and AST 121) without synthetic dysfunction. Notably, inflammatory markers were benign (WBC 6.5) and renal function was preserved (Cr 1.2). Electrocardiogram (ECG) demonstrated diffuse ST segment elevation with associated PR depression. Chest X-Ray (CXR) noted a large globular heart without significant pulmonary infiltrate.

**Fig. 1:**
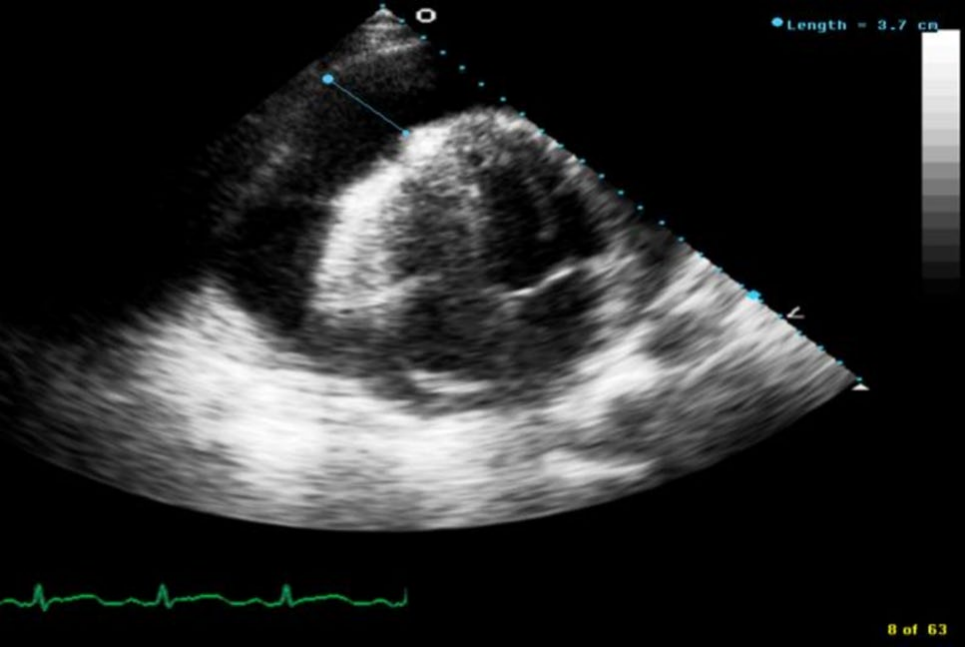
Transthoracic echocardiogram (TTE) in an apical four-chamber view demonstrating a large circumferential pericardial effusion with maximum diameter of 37 mm

**Fig. 2:**
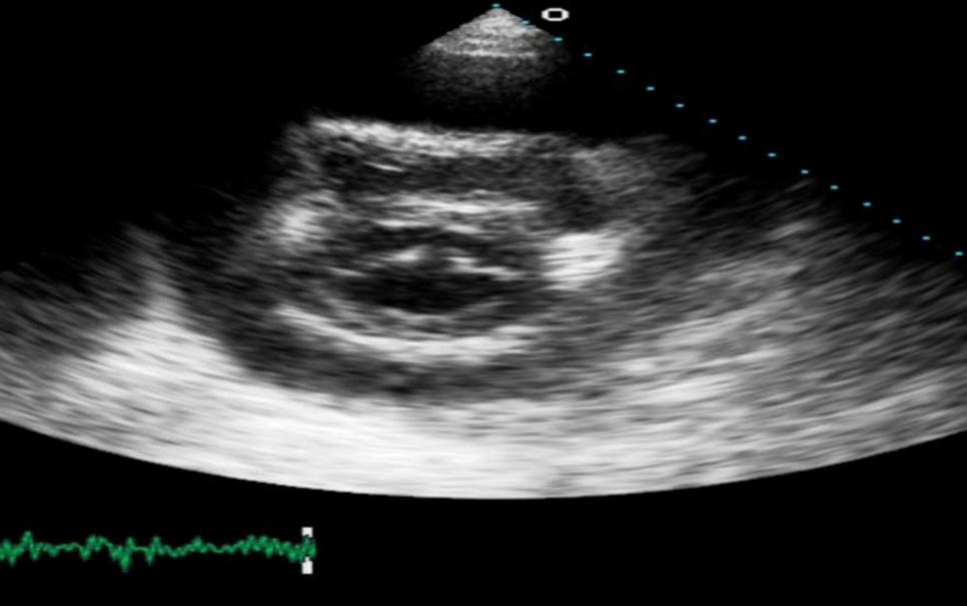
Transthoracic echocardiogram (TTE) in parasternal short axis view at level of mitral valve demonstrating diastolic collapse of right ventricular free wall secondary to large compressive pericardial effusion

**Fig. 3:**
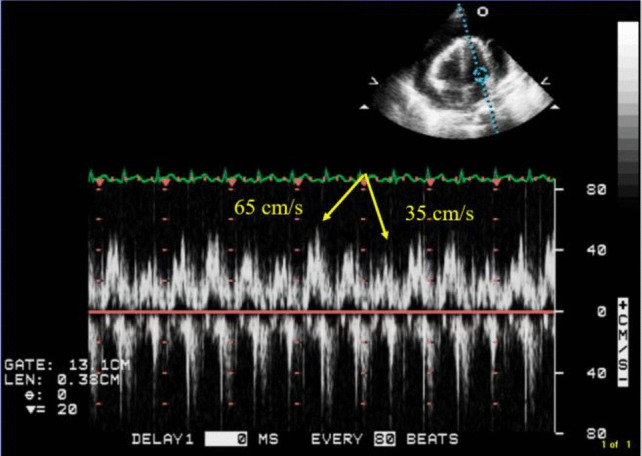
Transthoracic echocardiogram in an apical four-chamber view with doppler at mitral valve demonstrating variation in mitral inflow velocities with respiration

**Fig. 4:**
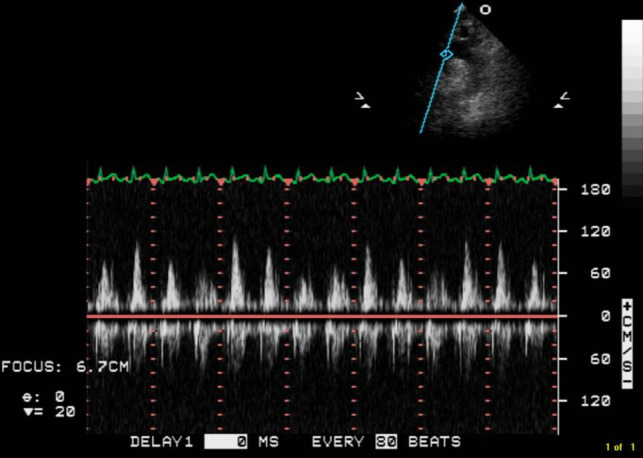
Transthoracic echocardiogram (TTE) in suprasternal view demonstrating exaggerated fall in systemic arterial flow velocities with inspiration—consistent with pulsus paradoxus

Transthoracic echocardiogram (TTE) revealed a large circumferential effusion (maximum thickness 37 mm) with visible fibrin stranding (Fig. 1). Diastolic collapse of the right ventricle (RV) was visible in the parasternal short axis view (Fig. 2) and doppler imaging noted respiration-induced variation in mitral valve flow (65 cm/s on expiration versus 35 cm/s on inspiration)—findings consistent with echocardiographic tamponade physiology (Fig. 3). Echocardiographic evidence of pulsus paradoxus was shown in the suprasternal view with exaggerated fall in systemic arterial flow velocities on inspiration (Fig. 4).

**Fig. 5:**
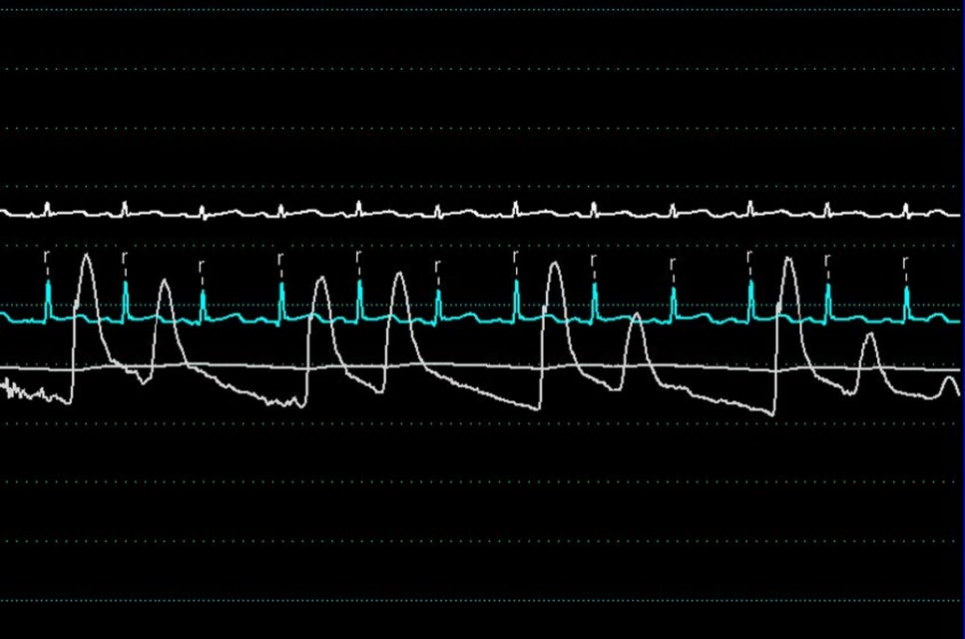
Femoral pressure tracing (white) demonstrating respiratory variation (pulsus paradoxus)

**Fig. 6:**
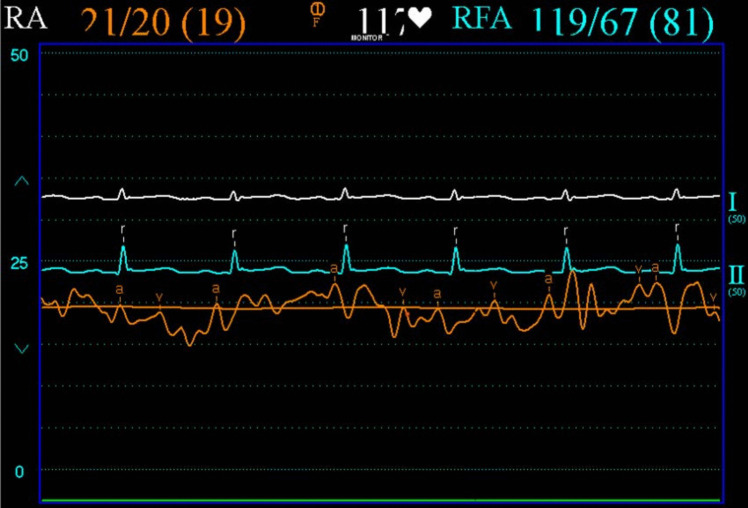
Right heart (orange) pressure tracing demonstrating elevated mean right atrial pressure and exaggerated ‘x’ descent

Right heart and femoral arterial pressure tracings were performed. Femoral artery pressures confirmed the presence of pulsus paradoxus (Fig. 5). Mean arterial pressures were elevated across the float with diastolic equalisation—RA pressures 21/20 (mean 19) mm Hg (normal range 1—5 mm Hg) with an exaggerated ‘x’ descent due to rapid RV filling (Fig. 6), RV diastolic pressures 37/17 (mean 19) mm Hg, pulmonary arterial pressure 37/16 (mean 25) mm Hg and pulmonary capillary wedge pressure (PCWP) 22 mm Hg. RA, RV and PA oxygen saturations were normal.

**Fig. 7:**
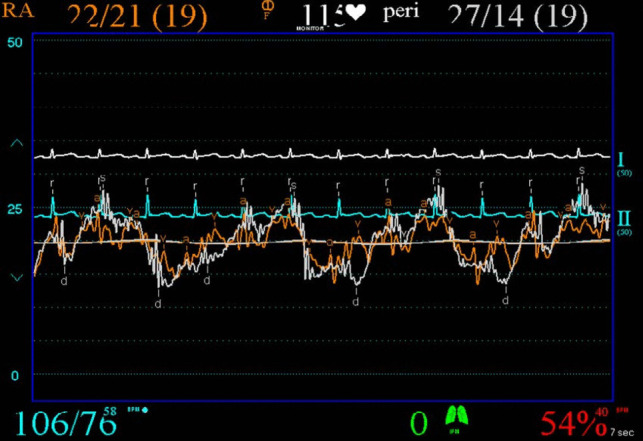
Right atrial (orange) and intra-pericardial (white) pressure tracings pre-pericardiocentesis demonstrating elevated right atrial and intra-pericardial pressures

Pericardiocentesis was performed along with simultaneous pericardial and RA pressure measurements (Fig. 7). Intra-pericardial pressure was significantly elevated with mean of 19 mm Hg (normal − 5 to 0 mm Hg). The overall picture shows systemic hypotension with pulsus paradoxus, elevated left atrial pressures as evident by the raised PCWP, elevated right heart pressures, and elevated intra-pericardial pressure—consistent with cardiac tamponade.

**Fig. 8:**
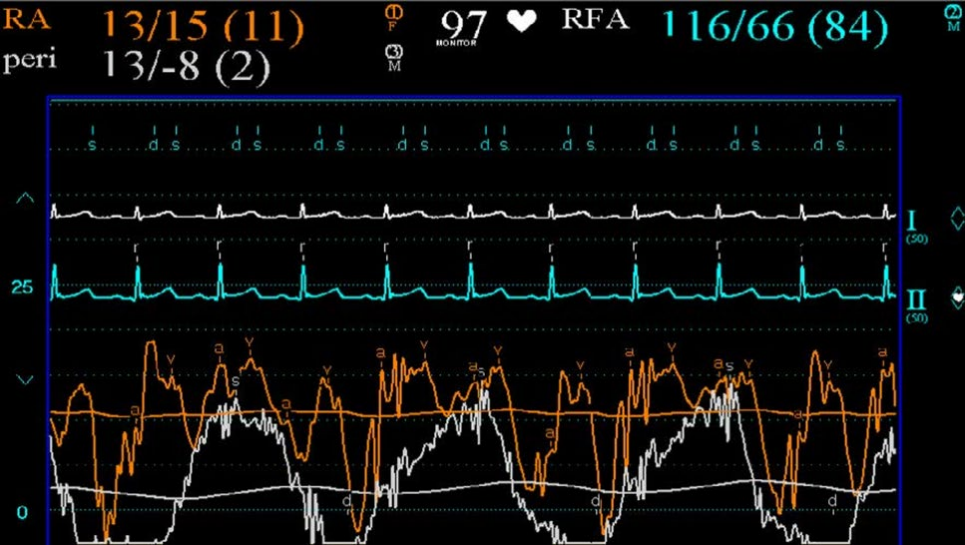
Right atrial (orange) and intra-pericardial (white) pressure tracings post pericardiocentesis demonstrating normalisation of intra-pericardial pressure along with persistently raised right atrial pressure and steep right atrial ‘y’ descent

Pericardiocentesis with immediate drainage of 900 mL of turbulent fluid resulted in an improvement in RA pressure (Fig. 8). Mean RA pressure fell from 19 mm Hg to 11 mm Hg. There was however a prominent ‘x’ descent and steep ‘y’ descent characteristic of constrictive pericarditis (Fig. 8). There was a reduction in pulsus pressure but not complete resolution. Failure of the RA pressure to drop by > 50% or to below 10 mm Hg and the steep ‘y’ descent are consistent with a diagnosis of ECP.

**Fig. 9:**
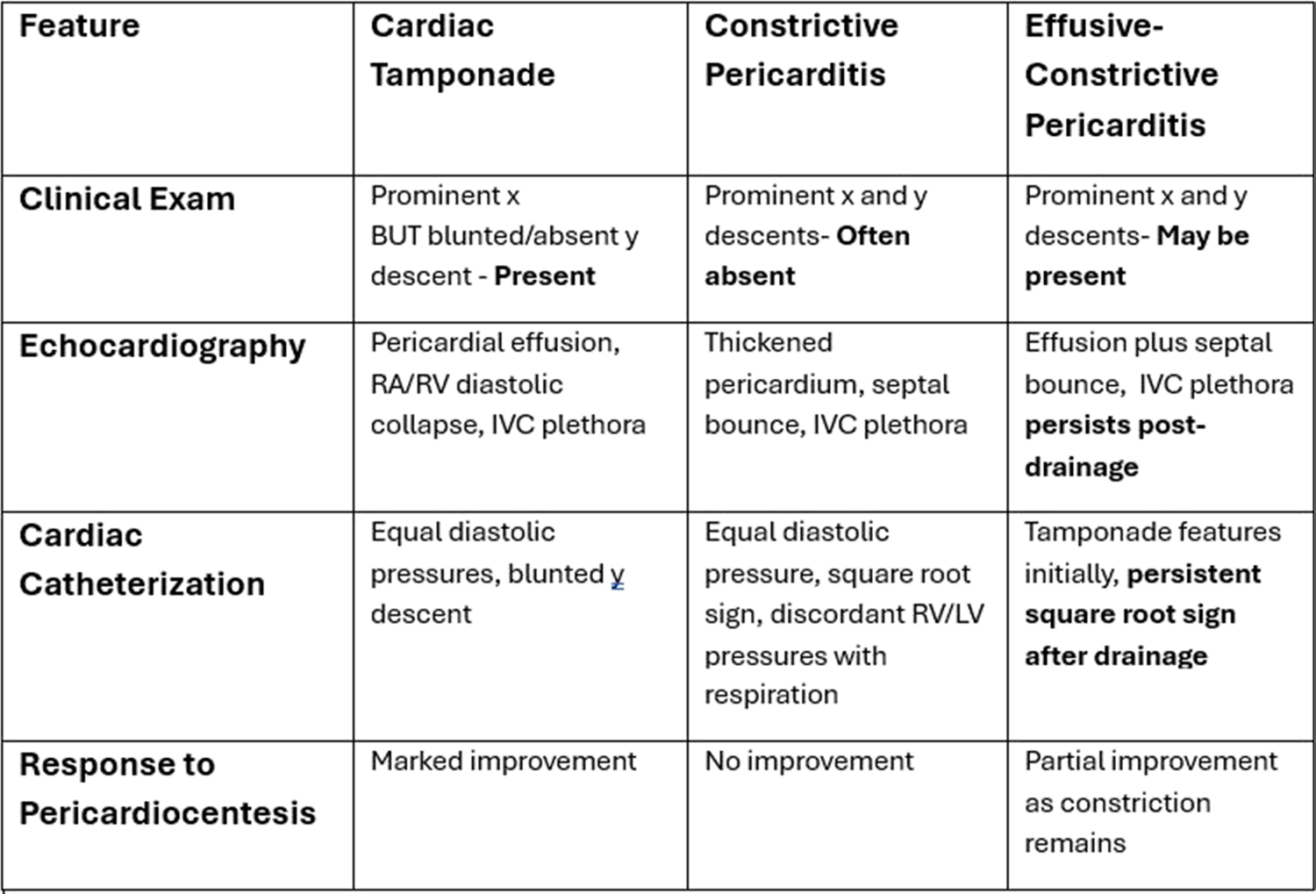
Table comparing the features of cardiac tamponade, constrictive pericarditis and effusive constrictive pericarditis

Pericardial fluid analysis was consistent with an exudative effusion. Fluid protein was 9.3 g/dL (serum sample 7.7 g/dL). Fluid lactate dehydrogenase (LDH) and serum LDH were 1216 U/L and 275 U/L respectively. There were 160 white blood cells (WBCs) in the pericardial fluid of which 84% were neutrophils—consistent with an inflammatory exudative effusion. Initial microscopy and gram staining of the pericardial fluid were negative. However, TB was subsequently cultured from the pericardial fluid despite previously negative blood, sputum and urine TB cultures. Furthermore, HIV serology was positive, and a viral load was 25,900 copies/mL. The patient was commenced on appropriate antiretroviral therapy (Fig. 9).

Ultimately due to persistent symptoms of right heart failure despite adequate decompression of the pericardial effusion, the patient was brought to the operating theatre for a pericardiectomy.

## Discussion

### Cardiac tamponade

Cardiac tamponade is a clinical syndrome defined by the inability of the heart to pump effectively secondary to a pericardial effusion. It is characterised clinically by hypotension, distant heart sounds and elevated JVP in the setting of an effusion [4]. The acuity of fluid accumulation determines risk of tamponade. In chronic slow-growing effusions, the pericardium becomes compliant, thus reducing the risk of tamponade in comparison to a rapidly accumulating acute effusion in a non-compliant pericardium [5, 6]. Echocardiographic features of tamponade are related to two key pathophysiologic principles—chamber collapse due to external compression during periods in the cardiac cycle of low intra-chamber pressure and ventricular interdependence [7]. One of the most sensitive early echocardiographic signs of tamponade is RA collapse during late diastole (when intra-atrial pressure is low) followed by early diastolic collapse of the RV (when intra-ventricular pressure is low) [8, 9]. Respiration-induced changes in intra-ventricular pressures drive swings in the interventricular septum giving rise to interventricular dependence and pulsus paradoxus. Tamponade physiology on echocardiogram was evident in this case including right ventricular free wall diastolic collapse (Fig. 2), respiration-induced changes in mitral inflow into the left ventricle (LV) (Fig. 3) and pulsus paradoxus (Fig. 4).

### Constrictive pericarditis

Constrictive pericarditis is characterised by an inability of the heart to fill secondary to a thickened non-compliant pericardium. It usually presents as a sub-acute or chronic right ventricular failure. Pericardial calcification is often, but not always, seen on CXR. Echocardiography can help distinguish constrictive pericarditis from clinically similar presentations such as restrictive myocardial disease or severe tricuspid regurgitation. Similar to cardiac tamponade, ventricular interdependence causing respiration-induced interventricular septum bowing is evident. However, retained mitral e’ velocities with reduced lateral mitral e’ velocities secondary to poor lateral excursion limited by the abnormal pericardium (known as annulus reversus) along with exaggerated mitral e′ velocities (known as annulus paradoxus) have been shown to reliably distinguish constrictive pericarditis from primary restrictive myocardial disease [10]. In cases of diagnostic uncertainty, invasive cardiac catheterisation remains the gold standard [8, 9]. Exaggerated ventricular pressure variation with respiration secondary to ventricular interdependence along with diastolic equalisation of pressures across the chambers (i.e. diastolic square root sign or dip-plateau pattern) are characteristic haemodynamic findings. These typical haemodynamic features were evident in our case post pericardiocentesis—indicative of underlying pericardial constriction.

### Effusive constrictive pericarditis (ECP)

ECP is a syndrome of combined compressive effusive and constrictive physiology. Historically, ECP was a rare diagnosis. Early studies report a prevalence of 1.3% in patients with pericardial disease [1]. A more recent retrospective study diagnosed ECP in 16% of patients undergoing pericardiocentesis [3]. Invasive cardiac catheterisation was required to confirm the diagnosis—a failure of RA pressure to drop by > 50% or to below 10 mm Hg post pericardiocentesis is diagnostic of ECP [1]. However, cardiac catheterisation is not performed routinely in cases of pericarditis or pericardial effusion. As such, cases of ECP were likely under-diagnosed in the past. In recent years, the identification of non-invasive echocardiographic features of constriction (and thus ECP if a compressive pericardial effusion is present) has increased diagnostic yield and should be sought routinely in patients presenting with pericardial effusion [3]. The underlying aetiology of the presenting pericardial effusion should also inform the possibility of ECP. Although ECP can occur with several pathologies, it frequently occurs with tuberculous pericarditis.

In patients with pericardial effusion who do not clinically improve post pericardiocentesis, ECP should be considered. Unfortunately, from the limited data available, ECP carries a poor prognosis. In early studies, greater than 50% of ECP patients required pericardectomy [1]. In a more recent systematic review of observational studies of ECP, the pericardiectomy rate was 65% [11]. The European Society of Cardiology (ESC) recommends that pericardiectomy only be performed for refractory cases in experienced centres given the difficulty of the surgical technique involved [12]. Many experts now advocate a trial of high-dose anti-inflammatory therapy prior to consideration of pericardiectomy in cases of constrictive disease post pericardiocentesis. However, the efficacy of anti-inflammatory agents such as colchicine in ECP has not been studied.

## Conclusion

ECP remains under-diagnosed among cases of pericardial effusion. The haemodynamics governing cardiac tamponade, constrictive pericarditis and ECP are complex. However, recent advances in echocardiography have improved non-invasive diagnostic yield. ECP should be suspected in cases of pericardial effusion without significant clinical improvement post-pericardiocentesis.
